# Perturbed Microbial Ecology in Myasthenia Gravis: Evidence from the Gut Microbiome and Fecal Metabolome

**DOI:** 10.1002/advs.201901441

**Published:** 2019-07-31

**Authors:** Peng Zheng, Yifan Li, Jing Wu, Hanping Zhang, Yu Huang, Xunmin Tan, Junxi Pan, Jiajia Duan, Weiwei Liang, Bangmin Yin, Fengli Deng, Seth W. Perry, Ma‐Li Wong, Julio Licinio, Hong Wei, Gang Yu, Peng Xie

**Affiliations:** ^1^ Department of Neurology The First Affiliated Hospital of Chongqing Medical University Chongqing 400016 China; ^2^ Institute of Neuroscience and the Collaborative Innovation Center for Brain Science Chongqing Medical University Chongqing 400016 China; ^3^ NHC Key Laboratory of Diagnosis and Treatment on Brain Function and Disease Chongqing 400016 China; ^4^ The M.O.E. Key Laboratory of Laboratory Medical Diagnostics the College of Laboratory Medicine Chongqing Medical University Chongqing 400016 China; ^5^ Social Medicine and Health Management Chongqing Medical University Chongqing 400016 China; ^6^ Department of Psychiatry College of Medicine SUNY Upstate Medical University Syracuse NY 13210 USA; ^7^ Precision Medicine Institute The First Affiliated Hospital Sun Yat‐sen University Guangzhou Guangdong 510080 China

**Keywords:** fecal microbiota transplantation, germ‐free mice, gut microbiome, metabolome, myasthenia gravis

## Abstract

Myasthenia gravis (MG) is a devastating acquired autoimmune disease. Emerging evidence indicates that the gut microbiome plays a key role in maintaining immune system homeostasis. This work reports that MG is characterized by decreased α‐phylogenetic diversity, and significantly disturbed gut microbiome and fecal metabolome. The altered gut microbial composition is associated with fecal metabolome changes, with 38.75% of altered bacterial operational taxonomic units showing significant correlations with a range of metabolite biomarkers. Some microbes are particularly linked with MG severity. Moreover, a combination of microbial makers and their correlated metabolites enable discriminating MG from healthy controls (HCs) with 100% accuracy. To investigate whether disturbed gut mcirobiome might contribute to the onset of MG, germ‐free (GF) mice are initially colonized with MG microbiota (MMb) or healthy microbiota (HMb), and then immunized in a classic mouse model of MG. The MMb mice demonstrate substantially impaired locomotion ability compared with the HMb mice. This effect could be reversed by cocolonizing GF mice with both MMb and HMb. The MMb mice also exhibit similar disturbances of fecal metabolic pathways as found in MG. Together these data demonstrate disturbances in microbiome composition and activity that are likely to be relevant to the pathogenesis of MG.

## 1 Introduction

Myasthenia gravis (MG) is an autoimmune disease characterized by variable muscle weakness, with the primary subtype understood to be due to antibodies binding to acetylcholine receptors (AchRs) at the neuromuscular junction (NMJ).^1^ Other subtypes exist with antibodies that bind to functionally related postsynaptic NMJ membrane molecules including muscle‐specific kinase (MuSK) and lipoprotein receptor–related protein 4 (LRP4),^1^ with all subtypes being autoimmune diseases with suspected defective immunomodulatory signaling.^2^, ^3^ The clinical symptoms of MG are diverse and overlap with other neurological disorders.^4^ Assisted antibody testing is relatively expensive, time consuming, not always available, and subject to a high rate of false negatives.^1^ Moreover, although conventional hormone and immunosuppressive therapy can improve the symptoms of most MG patients,^5^ the recurrence rate of MG is still quite high. Therefore, identification of new clinical diagnostics and biomarkers has the potential to be of great value for diagnosing, understanding, and treating MG.

The gastrointestinal tract is a complex ecosystem comprising a large number of resident microorganisms,^6^ which live symbiotically with the host and impact human nutrition, metabolism, and immune function.^7^, ^8^, ^9^ Previous studies have demonstrated altered gut microbiome to be associated with several complex diseases such as obesity, diabetes, and cancer.^10^, ^11^, ^12^ Recent emerging evidence shows that gut microbiome changes were linked with onset of disorders including stroke, epilepsy, Parkinson's disease, depression, and schizophrenia via the “microbiota‐gut‐brain” (MGB) axis.^13^, ^14^, ^15^, ^16^, ^17^ Thus it is becoming increasingly clear that the gut microbiome is a central regulator of host immune homeostasis,^9^, ^18^ whose dysregulation may participate in the onset of central (e.g., multiple sclerosis) and peripheral (e.g., rheumatoid arthritis) autoimmune diseases.^19^, ^20^ These findings compel us to determine whether MG, an immune‐mediated disease of the NMJ, is accompanied by altered microbial composition and activity.

To answer this question, we first compared the gut microbial communities of MG subjects and healthy controls (HCs), to evaluate whether altered gut microbiome composition was linked with status, severity, and/or diagnosis of MG. 16S ribosomal RNA (rRNA) gene sequencing based metagenomic methods can interrogate the microbial composition,^21^ but cannot provide the necessary quantitative functional annotation. Fecal metabolomics provides a complementary functional readout of microbial activity, and combining these two omics‐methods is a well‐established strategy for identifying potentially disease‐relevant gut microbes and their functional metabolites.^22^ Therefore, gas chromatography‐mass spectrometry (GC‐MS) was simultaneously used to capture the metabolic changes in the fecal samples obtained from MG. By integrating fecal microbiome and metabolomics findings, we sought to determine whether and how MG was associated with changes in microbial composition and function, and further identify potential biomarkers for MG. Furthermore, to assess whether gut microbiome disturbances may influence the onset of MG, fecal microbiota transplantation (FMT) into germ‐free mice was performed.

## 2 Results

### 2.1 Lower Within‐Sample Bacterial Diversity in MG Subjects

A total of 70 MG subjects and 74 HCs were recruited. The detailed characteristics of the recruited participants were shown in **Table** **1**. There were no significant differences in age or gender between the two groups. Using 16S ribosomal RNA gene‐sequencing, we identified 6778477 high‐quality reads in 144 fecal samples with an average length of 431.7. These reads were clustered into 895 Operational Taxonomic Units (OTUs) at 97% sequence similarity. The Venn diagram showed that 771 OTUs were shared between the MG and HCs groups, while 50 and 74 OTUs were unique to MG subjects and HCs, respectively (Figure S1a, Supporting Information).

**Table 1:** Detailed clinical characteristics of the subjects

Variables	MG	HC	*p* ^a)^
Sample Size	70	74	–
Female	39 (55.7%)	44 (59.4%)	0.652
Age—years^a)^	46.12 ± 1.54	44.86 ± 1.94	0.571
Duration of disease—years^b)^	5.70 ± 0.79	–	–
Thymic hyperplasia	23 (32.8%)	–	–
History of respiratory crisis	19 (27.1%)	–	–
Antibody test	23 (32.8%)	–	–
Anti‐AChR antibody (+)	15 (21.4%)	–	–
Immunosuppressive treatment	31 (44.3%)	–	–
Osserman classification			
I	31 (44.3%)	–	–
IIa	18 (25.7%)	–	–
IIb	21 (30.0%)	–	–
QMG^c)^	4.19 ± 0.55^b)^	–	–

^a)^ Two‐tailed student test for continuous variables (age), Chi‐square analyses for categorical variables (sex)

^b)^ Values are expressed as the mean ± standard error of mean

^c)^ Quantitative Myasthenia Gravis (QMG) test, QMG scores range from 0 to 39

Abbreviations: MG, myasthenia gravis; HC, healthy control.

**Figure 1:**
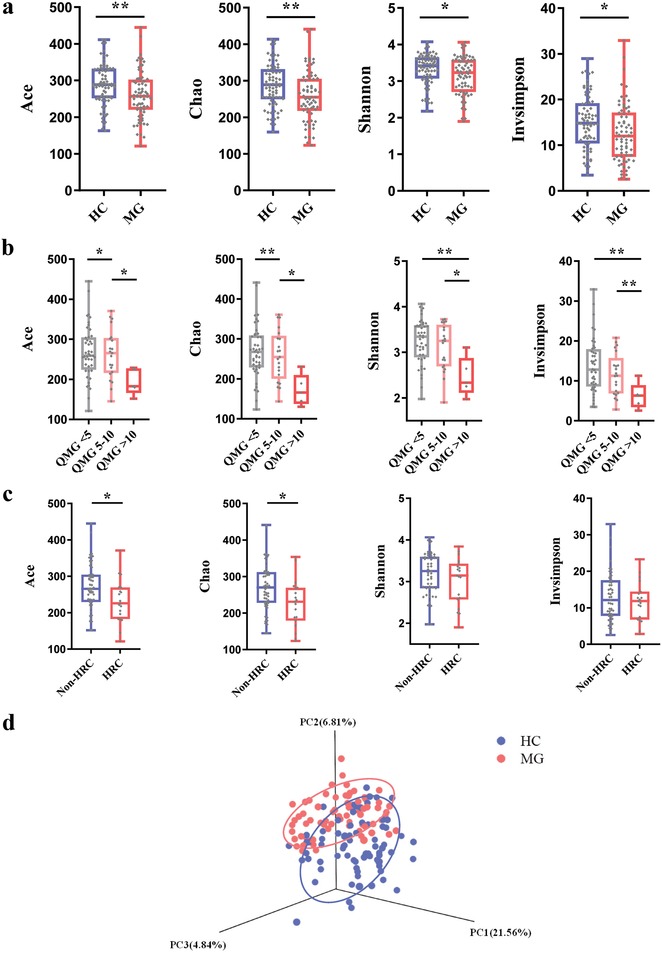
Gut microbial characteristics of myasthenia gravis (MG). a) α‐phylogenetic diversity analysis showing that MG subjects were characterized by lower microbial richness in four indexes (Ace, Chao, Shannon, and Invsimpson) relative to healthy controls (*n* = 74, HC; *n* = 70, MG). b) The MG subjects with different QMG scores showing different α‐phylogenetic diversity indexes (*n* = 70, MG). The four α‐ diversity indexes consistently decreased with the increases of QMG scores, highlighting a robust association between lower some α‐ diversity indexes and MG severity.(QMG < 5, *n* = 44; QMG = 5–10, *n* = 21; QMG > 10, *n* = 5). c) The MG subjects with history of respiratory crisis (HRC, *n* = 19) showing lower ACE and Chao indexes compared to the non‐history of respiratory crisis subjects (Non‐ HRC, *n* = 51). As the HRC is a hallmark of MG severity, this finding also confirms the association between α‐ diversity indexes and MG severity. d) At the operational taxonomic units (OTU) level, 3D principal co‐ordinates analysis showed that gut microbial composition of patients with MG was significantly different from that in healthy controls (*n* = 74, HC; *n* = 70, MG).

Next, the within‐sample (α)‐phylogenetic diversity indexes including microbial community richness (Chao, Ace) and diversity (Shannon, Invsimpson) were compared between the two groups. We found that both microbial richness and diversity were significantly different between MG and HCs subjects. Compared to HCs, the MG subjects were characterized by decreased Chao, Ace, Shannon, and Invsimpson indexes (**Figure** **1**a), suggesting decreased α‐phylogenetic diversity in MG.

### 2.2 Microbial Diversity as a Novel Diagnostic Tool for MG Severity

To evaluate whether α‐diversity correlates with MG severity, the MG patients were divided into three groups based on their quantitative myasthenia gravis (QMG) scores, and the indexes of α‐diversity were compared. We found that the four indexes of α‐diversity were substantially different among the three MG severity groups, showing that the four indexes consistently decreased with increased QMG scores (Figure 1b). Moreover, two α‐diversity indexes (Ace and Chao) of the MG patients with history of respiratory crisis (HRC) were also decreased compared to that in the MG patients without HRC (Figure 1c). These findings suggest a close association between lower α‐diversity indexes and MG severity. Consistent with this finding, while analyzing the correlation between α‐diversity indexes and key demographic characteristics, Shannon and Chao indexes showed negative correlation with QMG scores. However, other demographic variables such as age and BMI showed little correlation with α‐diversity indexes (Table S1, Supporting Information). These findings highlight that microbial diversity indexes may provide a novel clinical tool for assessing MG severity.

### 2.3 MG Subjects Harbor Different OTUs of Gut Bacteria versus HCs

To determine whether MG was associated with altered microbial composition, β‐diversity analysis was performed. 3D principal coordinates analysis revealed that the gut microbiota composition of MG subjects was significantly different from that of HCs (Figure 1d). Otherwise, control analyses showed that the HC or MG subjects were not clustered based on gender (Figure S1b, Supporting Information) or age (Figure S1c, Supporting Information). Microbial compositions for MG subjects were not significantly clustered based on disease subtype (Figure S1d, Supporting Information), medication/treatment history (Figure S1e, Supporting Information), or AChR antibody status (Figure S1f, Supporting Information). Together these results suggest that global microbial phenotypes were not significantly influenced by any of these other potentially confounding variables.

**Figure 2:**
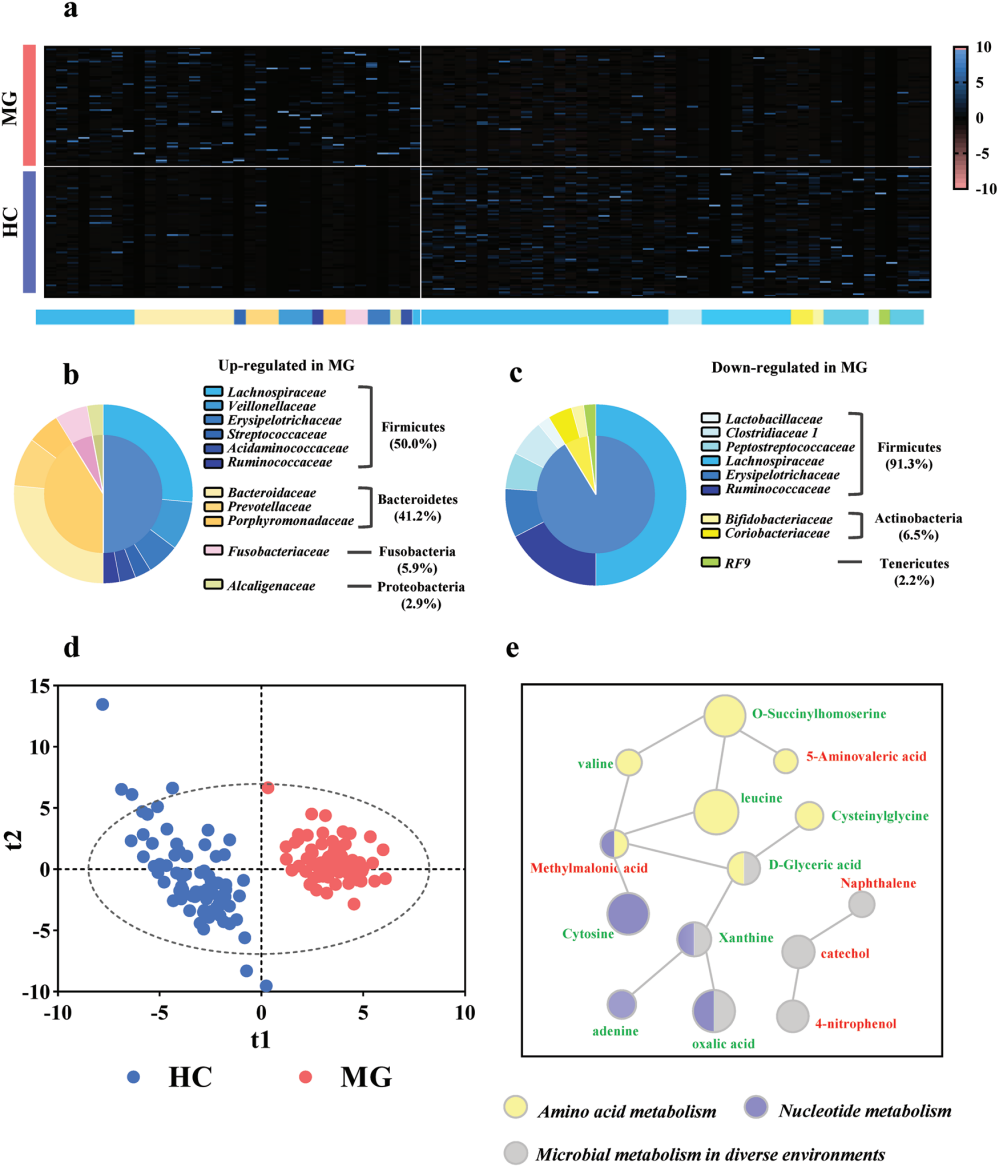
Gut microbial characteristics of MG. a) Using linear discriminant analysis (LEfSe), 80 differential OTUs responsible for discriminating the gut microbiota in MG and HCs subjects were identified. Heatmap of the 80 discriminative OTUs abundances between MG subjects and healthy controls; b,c) 34 upregulated OTUs in MG are arranged on the left, while 46 decreased OTUs are arranged on the right. The taxonomic assignment of each OTU is provided on the above of Heatmap. Most of the upregulated OTUs belongs to Firmicutes (50.0%), Bacteroidetes (41.2%) and Fusobacteria (5.9%), while downregulated OTUs mainly belongs to Firmicutes (91.3%) and Actinobacteria (6.5%). d) The Partial least‐squares discriminant analysis (PLS‐DA) scores plot showing a clear discrimination between MG subjects and HCs. e) The key disturbed metabolic pathways in MG. The red label indicates the increased metabolites, while the green label indicates the decreased metabolites in MG subjects relative to HCs. The *p*‐value of each metabolite is represented by the circle area. IPA software analysis showed that MG was mainly associated with disturbances of amino acid metabolism, nucleotide metabolism and microbial metabolism in diverse environments. (*n* = 74, HC; *n* = 70, MG).

Subsequent linear discriminant analysis effect size (LEfSe) analysis identified 80 differential OTUs that were responsible for discriminating MG subjects from HCs (**Figure** **2**a; Table S2, Supporting Information). Compared to HCs, the MG subjects were characterized by 34 increased OTUs (Figure 2b), which mainly belonged to the bacterial taxonomic families Bacteroidaceae (9 OTUs), Lachnospiraceae (9 OTUs), Veillonellaceae (3 OTUs), and Prevotellaceae (3 OTUs). The 46 OTUs that were decreased in MG subjects relative to HCs mainly belonged to the bacterial families Lachnospiraceae (23 OTUs), Ruminococcaceae (8 OTUs), Erysipelotrichaceae (4 OTUs), Peptostreptococcaceae (3 OTUs), and Clostridiaceae_1 (3 OTUs) (Figure 2c). Overall the 80 discriminative OTUs primarily belonged to the phyla Firmicutes (59/80, 73.75%), Bacteroidetes (14/80, 17.5%), and Actinobacteria (3/80, 3.75%). Similarly, compared with HC subjects, the abundances of Fusobacteria (MG vs HC, 141.65 ± 46.95 vs 9.59 ± 6.63; *p* < 0.01) and Bacteroidetes (MG vs HC, 2920.02 ± 379.61 vs 1323.36 ± 384.51; *p* < 0.01) were increased in MG subjects, while those of Actinobacteria (MG vs HC, 463.58 ± 109.57 vs 1282 ± 175.33; *p* < 0.001) were decreased.

### 2.4 MG Subjects Display Changes in the Fecal Metabolome

The fecal metabolome is considered a functional readout of the gut microbiome. We found that the fecal metabolic phenotype of MG was significantly different than that of HCs (Figure 2d), with 30 fecal metabolites identified as being responsible for discriminating between the two groups (Table S3, Supporting Information). Compared to HCs, MG was characterized by decreased levels of 16 metabolites, as well as 14 increased metabolites. Functional clustering analysis showed that the majority of differentially expressed metabolites were consistently involved in amino acid metabolism (valine, leucine, cysteinylglycine, methylmalonic acid, 5‐aminovaleric acid, d‐Glyceric acid, and O‐Succinylhomoserine); nucleotide metabolism (xanthine, cytosine, adenine, methylmalonic acid, and oxalic acid); and microbial metabolism in diverse environments (d‐Glyceric acid, naphthalene, catechol, 4‐nitrophenol, oxalic acid, and xanthine) (Figure 2e).

### 2.5 Correlations of Gut Microbial OTUs with Metabolites and Some Clinical Characteristics of MG

**Figure 3:**
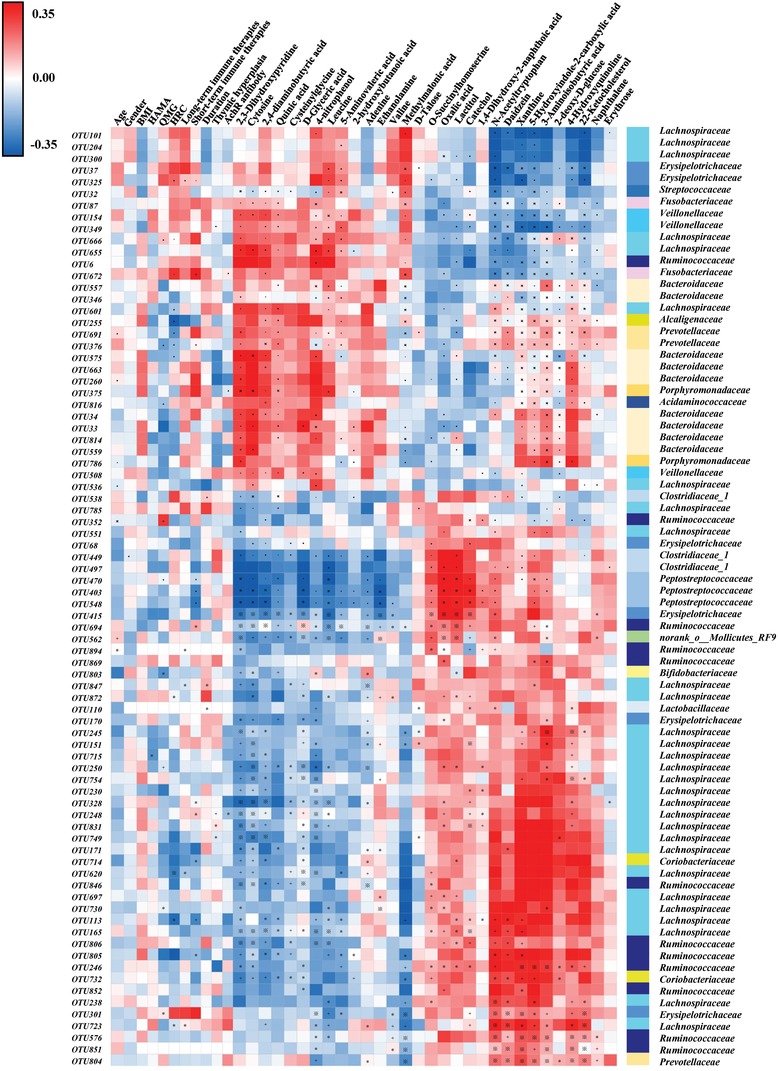
Associations of gut microbial species with fecal metabolites and clinical indexes. Heat map of the Spearman's rank correlation coefficient of 80 gut microbial OTUs and 30 metabolites as well as 11 clinical indexes. Red squares indicate positive associations between these microbial species and metabolites or clinical indexes; blue squares indicate negative associations. The statistical significance was denoted on the squares (*p < 0.05; +*p* < 0.01; ※*p* < 0.001). 31 of 80 differential microbial variances (38.75%) were significantly associated with differential metabolites (*p* value < 0.001 and correlation coefficient were ≥0.35 or ≤−0.35, tested by Spearman correlation). Moreover, some gut microbial OTUs were slightly associated with some clinical parameters such as gender, HAMA, long‐term immune therapies, duration, thymic hyperplasia and well‐established acetylcholine receptor (AchR) antibody, but highly linked with indicators of MG severity (QMG score, HRC, and short‐term immune therapies). Abbreviation: BMI, body mass index; HAMA, Hamilton anxiety scale; QMG, quantitative myasthenia gravis; HRC, history of respiratory crisis.

**Figure 4:**
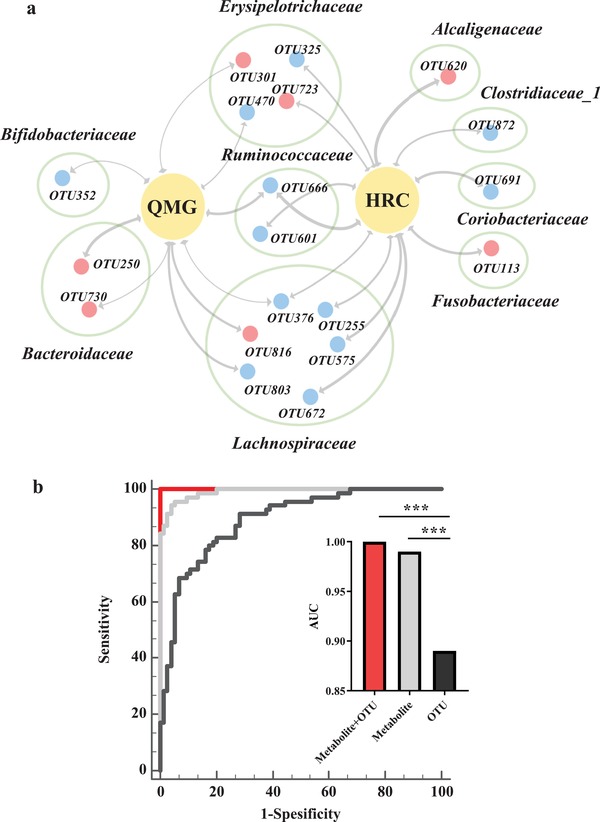
Microbial and metabolite markers for MG. a) the association between MG severity and microbial OTUs. 21 OTUs were significantly linked with MG severity (QMG score and HRC). Majority of these OTUs mainly belonged to Lachnospiraceae (6 OTUs), Erysipelotrichaceae (4 OTUs), and Bacteroidaceae (2 OTUs) at family level. Red dots indicate the upregulated microbial OTUs, while blue dots indicate the downregulated OTUs in MG related to HCs. The thickness of the line represents the size of the *p*‐value (*p* < 0.05). b) diagnostic markers for MG: Combination of 4 microbial marker (Clostridiaceae 1, Lachnospiraceae, Erysipelotrichaceae, and Bacteroidaceae) and 6 metabolites (leucine, cytosine, oxalicacid, *N*‐Acetyltryptophan, d‐Glyceric acid, and xanthine) can distinguish the MG from HCs with an area under the curve (AUC) of 1 (accuracy of 100%), which was significantly higher than that of separate microbial or metabolic markers.

To further explore the relationships between disturbances of gut microbiome, fecal metabolome, and MG clinical symptomology, correlation analysis was performed. We found that the differential bacterial OTUs were generally associated with differential metabolites (**Figure** **3**), with 38.75% (31/80 OTUs) of altered bacterial OTUs showing significant correlations with a range of metabolite biomarkers (associated with more than one metabolite, *r* > ± 0.35, *p*‐value < 0.001). These findings demonstrated that MG was simultaneously characterized by disturbed gut microbiome and fecal metabolome. Additionally, we found that some gut microbial OTUs were slightly associated with some clinical parameters such as gender, hamilton anxiety scale (HAMA), long‐term immune therapies, duration, thymic hyperplasia, and well‐established acetylcholine receptor (AchR) antibody (Figure 3). Surprisingly, we found that 19 OTUs were highly linked with indicators of MG severity (QMG score and history of respiratory crisis) (Figures 3, and 4a). These OTUs mainly belonged to Lachnospiraceae (6 OTUs), Erysipelotrichaceae (4 OTUs), and Bacteroidaceae (2 OTUs) at family level (**Figure** **4**a).

### 2.6 A Panel of Microbial and Metabolic Markers Identifies MG with 100% Accuracy

To identify and quantify the potential diagnostic capabilities of our newly identified microbial and metabolic biomarkers in MG, a binary logistic regression analysis for the differential 31 OTUs and 8 significantly correlated metabolites was performed. We found that the most significant deviations between MG and HC subjects could be described by 4 OTUs belonging to Clostridiaceae 1 (OTU562), Lachnospiraceae (OTU672), Erysipelotrichaceae (OTU723), and Bacteroidaceae (OTU749) at the taxonomic family level, as well as 6 significantly correlated metabolites (leucine, cytosine, oxalic acid, *N*‐Acetyltryptophan, d‐Glyceric acid, and xanthine). This marker panel in combination enabled discriminating MG from HC with 100% accuracy, which was significantly higher than that of separate microbial or metabolic markers (Figure 4b).

### 2.7 Transplantation of MG Microbiota Induces Impaired Locomotion Ability in GF Recipient Mice

**Figure 5:**
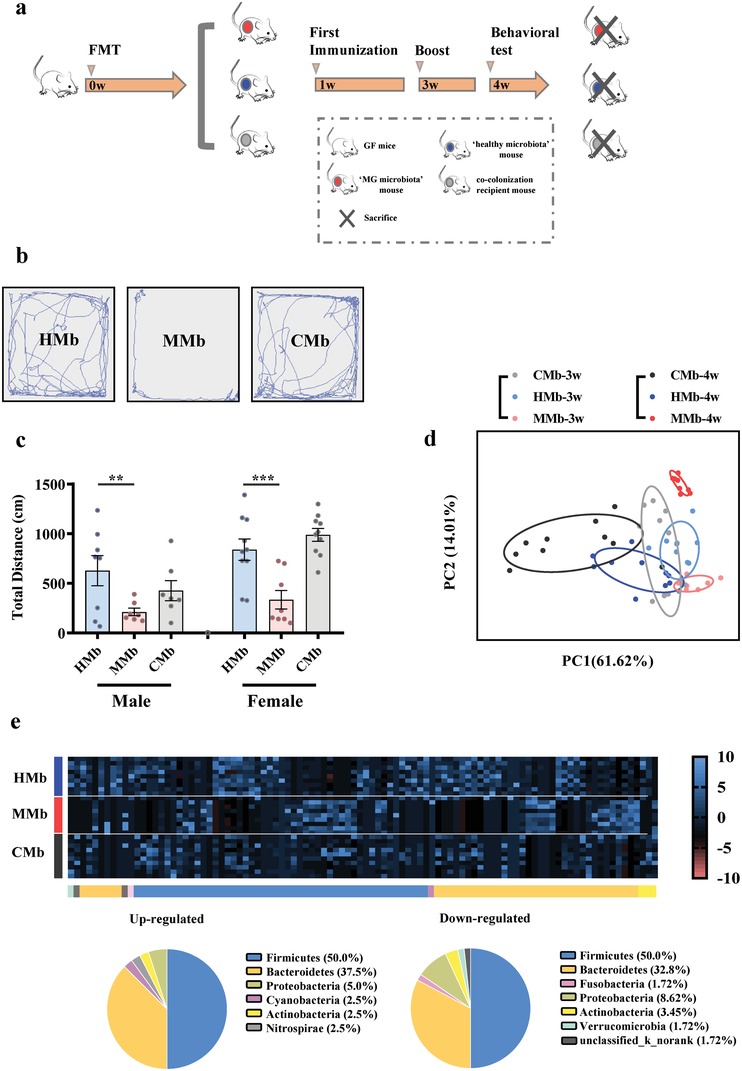
a) The workflow of fecal microbiota transplant (FMT) experiment. Briefly, adult male a germfree (GF) Kunming mice were colonized with fecal samples derived from MG patients or healthy controls, thus generating the “MG microbiota” recipient mice(MMb) and “healthy microbiota” recipient mice (HMb). Moreover, to further verify the effect of gut microbiome on behavior, an independent GF mice group was simultaneously colonized with fecal samples from both MG and HC to generate the cocolonization recipient mice. Three groups were immunized twice at second and fourth weeks. The Openfield test (OFT) was performed at fifth weeks. To verify the generality of the results, we conducted two separate experiments using both female and male GF mice. b,c) Representative motion tracks for a “MG microbiota” mouse (MMb), a “healthy microbiota” mouse (HMb) and a cocolonization recipient mouse (CMb). The total motion distance of “MG microbiota” was substantially decreased compared to of that in “healthy microbiota” recipient mice, and showed no significant differences with that in cocolonization recipient mice. The consistent results were observed while using the male and female GF mice in the two independent experiments (*n* = 8/HMb male group, *n* = 7/MMb male group, *n* = 7/CMb male group; *n* = 10/HMb female group, *n* = 8/MMb female group, *n* = 10/CMb female group). Data presented as means ± standard error of mean. * *p* < 0.05, **p < 0.01, ***p < 0.001 by one‐way ANOVA. d) At the operational taxonomic units (OTU) level, principal component analysis showed that gut microbiota composition of “MG microbiome” recipient mice was significantly different from that in healthy controls. e) Using linear discriminant analysis (LEfSe), 98 differential OTUs responsible for discriminating the gut microbiota in MMb and HMb mice were identified. Heatmap of the 98 discriminative OTUs abundances between MG subjects and healthy controls; 40 upregulated OTUs in MMb mice are arranged on the left part of the image, and 58 decreased OTUs are arranged on the right part. The taxonomic assignment of each OTU is provided on the above of Heatmap. (*n* = 10, HMb; *n* = 8, MMb; *n* = 10, CMb).

To assess whether and how the MG microbiome (MMb) might influence the development of MG, FMT experiments were performed (**Figure** **5**a). In these experiments, germ‐free (GF) mice were initially colonized with MMb or healthy microbiota (HMb) or both MMb and HMb (CMb), and then immunized using classic modeling methods.^23^, ^24^ The microbial phenotype of FMT samples were representative of their respective full samples (Figure S2, Supporting Information). We next tested whether MMb affected MG‐related locomotion using the open‐field test (OFT), 4 weeks after FMT. The representative motion tracks for a MMb mouse, a HMb mouse, and a CMb mouse are shown in Figure 5b. The total distance traveled was substantially decreased in MMb mice compared to HMb mice, but showed no significant difference compared to the CMb mice (Figure 5c). The same behavioral results were obtained using both male and female GF FMT mice, suggesting a generalizability of this finding to both sexes. Taken together, we found that, under the same immune conditions, colonization of GF mice with MMb resulted in impaired locomotion ability compared to colonization with HMb. This effect could be reversed by cocolonizing GF mice with both MMb and HMb (CMb mice), suggesting that the gut microbiome may contribute to the development of MG.

Previous clinical and animal experiments have demonstrated altered cytokine levels as hallmark of MG. Therefore, we compared cytokine levels in serum and intestinal tissue among the three mouse groups. We found that serum levels of TNF‐α, IFN‐γ, and IL‐10 were significantly increased in MMb group compared to the HMb group (Figure S3a,b,d, Supporting Information). These increased cytokines observed in MMb mice could be reversed by coadministration of HMb, as the levels of these three cytokines in the CMb group were comparable to that in HMb group. Moreover, compared to the HMb group, the intestinal tissue levels of TNF‐α, IFN‐γ, and IL‐6 were also significantly upregulated in MMb group; and these changes were reversed to baseline in the CMb group (Figure S3e–g, Supporting Information). Here, we observed increased inflammatory cytokines in the MMb group, further supporting a possible role of the gut microbiome in the development of MG.

### 2.8 Characterizing Gut Microbiome Alterations in “MG microbiota” Recipient Mice

To investigate whether the microbial characteristics of MG patients were reproduced in the MMb mice, 16S rRNA gene sequencing was performed to characterize the fecal microbial community of the three experimental groups at 3 and 4 weeks after FMT. Importantly, the distinct microbial communities found in MG patients compared to HCs were reproduced in their respective FMT, MMb, and HMb mice (Figure 5d). Interestingly but not unexpectedly, we found that the samples originating from the CMb group shared characteristics of both the MMb and HMb groups, which may account for its ability to reverse the impaired locomotion and increased inflammatory cytokines observed in MMb mice. Using the fecal samples obtained at 4 weeks after FMT, 98 OTUs were identified that were responsible for discriminating the MMb and HMb groups (Figure 5e). These differential OTUs are mainly assigned into Firmicutes (49/98, 50.0%), Bacteroides (34/98, 34.7%), Actinobacteria (3/98, 3.1%), which were highly similar to the differences observed in the gut microbiomes between MG patients and healthy controls. This finding suggests that the key microbial characteristics of MG patients were maintained in the MMb mice. 54 of 98 differential OTUs between MMb and HMb group were reversed in the CMb group at different degree (Figure 5e; Table S4, Supporting Information).

**Figure 6:**
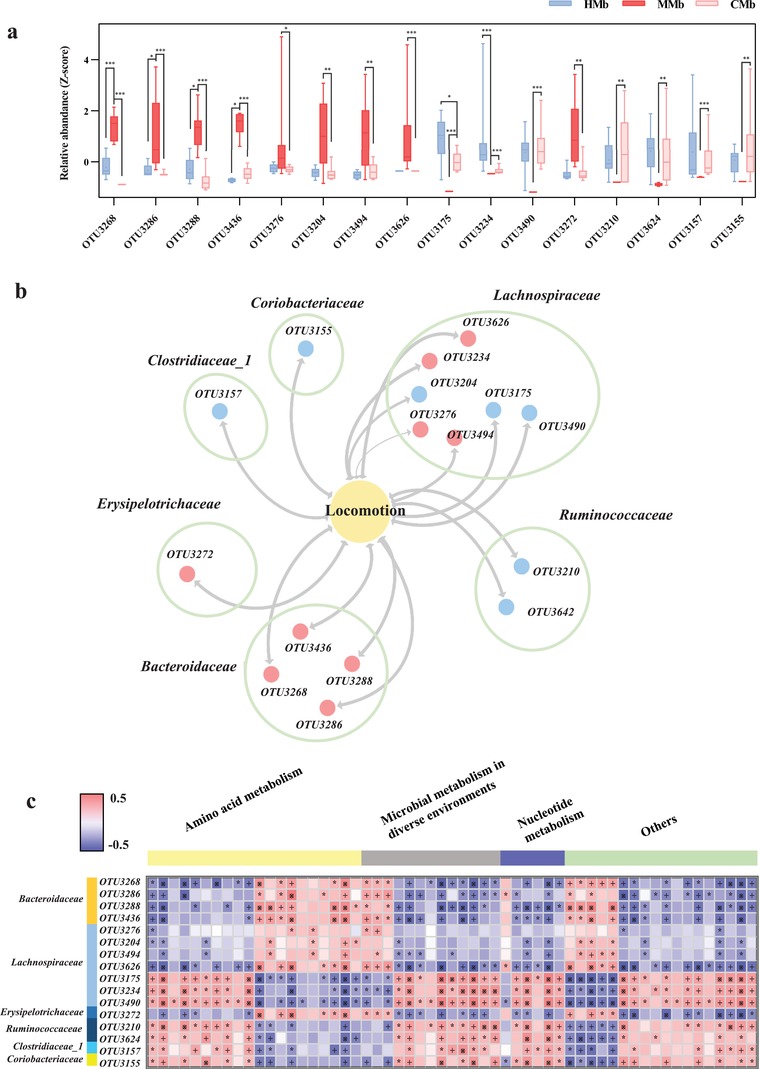
Gut microbial characteristics of MMb mice. a,b) 16 of 54 reversed OTUs mainly belonging to Lachnospiraceae (7 OTUs), Bacteroidaceae (4 OTUs), and Ruminococcaceae (2 OTUs) were linked with the total motion distance in the OFT (*n* = 10, HMb; *n* = 8, MMb; *n* = 10, CMb). This finding was highly similar with what had been observed in analysis of correlation between altered microbes and MG severity. Red dots indicate the upregulated microbial OTUs, while blue dots indicate the downregulated OTUs in MMb mice related to HMb mice, the thickness of the line represents the size of the *p*‐value (*p* < 0.05). c) Associations of gut microbial OTUs with fecal metabolites. Heat map of the Spearman's rank correlation coefficient of 16 gut microbial OTUs and 57 metabolites. The MMb mice was characterized by changes of amino acid metabolism, nucleotide metabolism and microbial metabolism in diverse environments. Red squares indicate positive associations between these microbial OTUs and metabolites; blue squares indicate negative associations (*n* = 9, HMb; *n* = 8, MMb). The statistical significance was denoted on the squares (*p < 0.05; +*p* < 0.01; ※*p* < 0.001).

Significantly, 16 of 54 reversed OTUs mainly belonging to Lachnospiraceae (7 OTUs), Bacteroidaceae (4 OTUs), and Ruminococcaceae (2 OTUs) were not only linked with the total motion distance in the OFT (**Figure** **6**a,b), but also associated with disturbances of fecal metabolomics which were mainly involved in disturbances of amino acid metabolism, nucleotide metabolism, and microbial metabolism in diverse environments (Figure 6c). The disturbed metabolic pathways in the MMb mice were consistent with those in MG patients. In sum, our findings demonstrate that colonization of GF mice with MMb resulted in impaired locomotion ability. These MMb mice reproduced the key fecal microbial and metabolic characteristics of MG patients, suggesting the gut microbiome may contribute to the development of MG by modulating host metabolism.

## 3 Discussion

Foremost in this study, we found that the combination of fecal microbial and metabolic markers can discriminate the MG subjects from healthy controls with an astonishing 100% accuracy, highlighting the potential of this approach as a novel diagnostic biomarker of MG. Clinically, 20% of MG patients suffer from the acute respiratory failure requiring mechanical ventilation (history of respiratory crisis), which results in considerably morbidity and mortality. Thus identification of biomarkers for MG severity, especially history of respiratory crisis, is of great clinical value. Autoantibodies such as AChR antibodies are the classical diagnostic biomarker of MG, but fail to reflect disease severity.^25^ Here, we identified a panel of microbes correlated with aspects of MG severity (e.g., severity score, history of respiratory crisis, and requirement of short‐term immune therapies). Our findings demonstrate the potential strengths and advantages of identifying markers for MG severity through non‐invasive analysis of the fecal gut microbiome and metabolomics.

We also demonstrated that MG was associated with microbial ecology perturbation with probable effects on both metabolism and function in human subjects. Some microbes were specifically correlated with MG severity. The combination of microbial markers and correlated metabolites enabled discriminating MG from HC subjects with high accuracy. Moreover, in translational experiments, colonization of GF mice with MMb resulted in impaired locomotion ability, upregulation of host inflammatory cytokines, and disturbed fecal metabolic pathways. Our findings provide novel insights by which we might better understand the pathogenesis of MG, and develop novel markers for MG.

We found that the microbial composition of MG is significantly different from that of HC subjects. This finding suggests that dysbiosis of gut microbiota may be associated with neuromuscular junction diseases, which is a new advance in investigations of how the gut microbiome shapes nervous system. We found that the microbial composition of MG was associated with lower α‐diversity compared to HCs. Generally speaking, a high α‐diversity is considered evidence of a “good” health status.^6^ Here, the lower α‐diversity in MG subjects suggests an abnormal microbial status compared to apparently healthy controls. Consistent with this finding, we found that MG subjects were mainly characterized by decreased OTUs belonging to Lachnospiraceae (23 OTUs) and Ruminococcaceae (8 OTUs). The Lachnospiraceae and Ruminococcaceae are two of the most abundant families from Clostridiales, and have been associated with the maintenance of gut health.^26^, ^27^ Consistent with lower α‐diversity in MG relative to HCs, these characteristic changes suggest abnormal microbial status in MG. Further metagenomic studies should be performed to identify the key bacterial taxa responsible for MG. Furthermore, we found that some microbes were linked with acetylcholine receptor (AchR) antibody, a well‐established biomarker for MG, and thymic hyperplasia, suggesting the gut microbiome may also influence the onset of MG through classical pathogenic pathways.

Furthermore, we found that MG was also associated with disturbances of fecal metabolomics. Significantly, the altered bacterial OTUs showed a high correlation with a range of metabolite biomarkers. These interesting findings suggest that the gut microbiome may affect the occurrence of MG through metabolic pathways including amino acid, nucleotide, and microbial metabolism. This is a potentially new complement to the previous antibody‐mediated pathogenesis and diagnosis of MG. It should be acknowledged that if and how these metabolic pathways participate in the onset of MG remains largely unclear and requires further investigation. Some hypothetical scenarios supported by our data are as follows: i) these metabolites related to microbial metabolism may support the idea that disturbances of gut microbiome may be associated with MG. This finding supports the current understanding that the fecal metabolome is the functional readout of the gut microbiome; ii) Regarding nucleotide metabolism, two metabolites (cytosine and methylmalonic acid) that were altered in MG subjects suggest a correlation between disturbed pyrimidine metabolism and MG. Consistent with this finding, three other metabolites (xanthine, adenine, and oxalic acid) involving purine metabolism were also disturbed in MG. These findings suggest that disturbances of nucleotide metabolism are linked with MG through modulating oxidative stress, a concept that is further supported by previous studies reporting altered antioxidant status at the protein or metabolite levels,^28^, ^29^ an area that is worth future study.

FMT is a useful translational experimental model for assessing whether altered gut microbiome may be involved in the development of various diseases. Here, under matching immune conditions, we found that the total distance traveled of MMb mice was substantially decreased compared to that of HMb mice, and showed no significant differences with that of CMb mice. Additionally, we found that the MMb mice also had increased serum and intestinal tissue inflammatory cytokines such as TNF‐α and IFN‐γ, and these increased these cytokines returned to baseline levels in the CMb group. Consistent with our findings, previous clinical and animal studies have found increased serum TNF‐α and IFN‐γ levels in MG.^30^, ^31^ In addition, intervention with cytokines has been shown to alleviate MG severity.^32^ Significantly, the MMb mice reproduced the key fecal microbial and metabolic characteristics of MG patients. And the altered microbes observed in MMb mice were significantly associated with the host's metabolic signature and its locomotion behavior. These findings suggest that disturbances of gut microbiome may participate in the pathological mechanisms of MG.

There were some limitations that should be acknowledged. Although here we provide evidence strongly suggesting that the gut microbiome may participate in the pathogenesis of MG, further research is needed to determine with certainty whether this relationship is causal or incidental. Furthermore, additional research should be performed to identify the specific microbial species and their corresponding metabolites related to MG, which will help further define novel targets for MG therapy. In addition, whereas all recruited MG subjects were symptomatic for MG, they varied in their medication status, which could potentially shape their microbial compositions. However, we found that the global microbial phenotypes of MG subjects were not clustered by medication status (Figure S1e, Supporting Information), suggesting that this was not likely to be a significant confounding variable. Nonetheless, future longitudinal studies to collect samples before and after medication use should be performed to validate and extend these current findings, to better ascertain whether our findings here are potentially causal or incidental to MG, or related to treatment response. Third, the diagnosis of MG in some candidate subjects with typical MG symptoms was not able to be confirmed with antibody tests due to clinical inaccessibility. Instead, other diagnostic tests such as repetitive electrical nerve stimulation and muscle fatigue tests were used for diagnosis of MG. Future studies in recurring MG subjects with different antibody types should be performed to help identify the specific microbial markers associated with different MG subtypes. Finally, the clinical diagnostic potential of our identified microbial and metabolic markers should be confirmed with multicenter independent samples, and the specificity should be assured by inclusion of other neurological diseases.

Taken together, we provide evidence that MG is characterized by disturbances of fecal microbial community diversity, composition, and function. We found that MG subjects can be effectively discriminated from HC subjects with high accuracy, with our identified microbial and metabolite markers. With translational FMT experiments, we provided evidence suggesting that disturbances of the gut microbiome may contribute to onset of MG. Our findings provide new avenues by which to enhance our understanding of MG pathogenesis, and to develop novel diagnostic and therapeutic tools for MG.

## 4 Experimental Section

*Subject Recruitment and Sample Collection*: The study protocol was approved by the Ethics Committee of Chongqing Medical University. All participants signed a written informed consent before any procedure was performed. In total, 70 MG subjects and 74 HCs were recruited from the neurology department and medical examination center of the First Affiliated Hospital at Chongqing Medical University, respectively. MG was diagnosed and classified based on previous literature.^33^, ^34^ The quantitative myasthenia gravis (QMG) test was used to quantify the severity of MG (scores range from 0 to 39).^35^ MG subjects were classified as one of three subtypes including I (*n* = 31), IIa (*n* = 18), and IIb (*n* = 21) based on clinical symptoms. The HAMA was used to quantify the mental status of MG subjects.^36^ All participants did not take any antibiotics, probiotics, or prebiotics within one month prior to sampling. Fresh stool samples were collected from each participant and immediately frozen at −80 °C until further analysis.

*DNA Extraction, Polymerase Chain Reaction (PCR) Amplification, and Illumina MiSeq Sequencing*: The Illumina MiSeq sequencing protocol was performed as previously described.^37^ Briefly, microbial DNA was extracted from stool samples using the QIAampDNA Stool Mini Kit (Qiagen, Hilden, Germany). The V3–V4 regions of the bacterial 16S rRNA gene were amplified by PCR using primers 338F (5′‐ACTCCTACGGGAGGCAGCA‐3′) and 806R (5′‐GGACTACHVGGGTWTCTAAT‐3′).^38^ PCR reactions were performed in triplicate 20 µL mixtures. Primers included an eight‐base sequence unique to each sample. Amplicons were extracted from 2% agarose gels and purified using the AxyPrep DNA Gel Extraction Kit (Axygen Biosciences, Union City, CA). Purified amplicons were quantified using QuantiFluor‐ST (Promega, US) and paired‐end sequenced (2 × 250) on an Illumina MiSeq platform according to the standard protocols.

*16S rRNA Gene Sequence Analysis*: Raw fastq files were demultiplexed, and quality‐filtered using QIIME (version 1.17, http://qiime.org/). The 250 bp reads were truncated at any site of more than three sequential bases receiving an average quality score <20. Reads shorter than 50 bp containing ambiguous base calls or barcode/primer errors were discarded. Chimeric sequences were checked by UCHIME (http://drive5.com/uchime) and removed from subsequent analyses. The remaining high quality sequences were clustered into operational taxonomic units (OTUs) at 97% similarity using usearch (version 7.0, http://drive5.com/uparse/). To assess adequacy of sequencing depth, rarefaction analysis was performed using the RDP Rarefaction tool based on the number of sequences and OTUs for each sample (http://sourceforge.net/projects/rdp‐classifier). α‐diversity was assessed using the species richness indexes (Ace and Chao) and species diversity indexes (Shannon, Invsimpson). Beta diversity were generated on the basis of unweighted UniFrac algorithms and reported according to principal coordinate analysis (PCoA).^39^ The key bacterial taxa responsible for discrimination between the two groups were identified using LEfSe.^40^

*Fecal Metabolome Analysis*: Gas chromatography‐mass spectrometry (GC/MS; Agilent 7890A/5975C) was used to perform the fecal metabolome analysis. The GC/MS 3D matrices comprised of peak indexes (RT‐m/z pairs), sample names (observations), and normalized peak area percentages were imported into SIMCA‐P+14.0 (Umetrics, Umeå, Sweden). Projection to latent structure discriminant analysis (PLS‐DA) was used to visually discriminate MG subjects from HCs. By analysis of PLS‐DA loadings, the differential metabolites responsible for discriminating between the two groups were identified with variable importance plot (VIP) values of greater than 1.0, and *p*‐values of less than 0.05. IPA software (Qiagen, Redwood City, CA, USA) was used to uncover the predicted molecular pathways and biological functions of the identified differentially expressed metabolites.

*FMT*: GF mice were obtained from the Department of Laboratory Animal Science of the Third Military Medical University (Chongqing, China). During the study, the mice were fed autoclaved chow and water ad libitum under a 12‐hour light/12‐hour dark cycle (lights on at 7:30 a.m.) and constant temperature of 21–22 °C and humidity of 55 ± 5%. All animal experiments followed the National Institutes of Health Guide for the Care and Use of Laboratory Animals and were approved by the Ethics Committee of Chongqing Medical University.

Fecal samples were selected from 70 MG patients (*n* = 8, male/female = 2/6, aged 15–70 years) and 74 healthy individuals (*n* = 8, male/female = 2/6, aged 15–70 years). The procedures of preparing the fecal samples for microbiota transplantation were as described in the previous studies.^41^, ^42^ Briefly, fecal samples were processed under anaerobic conditions. Each fecal sample (100 mg) was suspended in 1.5 mL of reduced sterile phosphate buffered saline and the pool was prepared with an equal volume of donor suspension. The MMb and HMb were propagated in separate autoclaved microisolator with individual sterile air supply, on autoclaved bedding and drinking water, and with sterilized standard chow ad libitum to prevent internalization of the gut microbiota. In each individual cage, MMb or HMb mice were propagated in different gnotobiotic isolator. To further verify the effect of gut microbiome on behavior, an independent GF mice group was simultaneously colonized with fecal samples from both MG and HC to generate the cocolonization recipient mice (CMb mice). Cecal samples were collected while killing mice and immediately frozen in liquid nitrogen and stored at −80 °C. To test the generality of FMT experiment, two independent experiments were performed using both male and female GF mice.

*Immunization of Mice with R97–116 Protein*: One week after FMT, the MMb, HMb, and CMb groups were subjected to construct the classic MG model (experimental autoimmune myasthenia gravis (EAMG) mouse model). The EAMG model was established as described previously.^24^ Briefly, all the mice were induced by subcutaneous injection into both hind footpads and back with 200 µL inoculum containing 50 µg R97–116 peptide (DGDFAIVKFTKVLLDYTGHI synthesized by Sangon Biotech (Shanghai) Co., Ltd) with complete Freund s adjuvant (Sigma‐Aldrich) 1 week after FMT and were boosted with 100 µL mixture along the same site 3 weeks after FMT.

*Behavioral Testing*: Based on previous studies, open‐field test (OFT) was used to determinate how the “MG gut microbiome” influence the host's locomotion ability.^43^ For this test, the mice were transferred to the experimental room for acclimation at least 1 h prior to behavioral testing. This behavioral test was carried out by observers who were blind to the animal genotypes between 8:00 and 17:00. The behavioral tests were videotaped and quantified by a video‐computerized tracking system (SMART, Panlab, Barcelona, Spain).^44^

*OFT*: All mice were individually tested in an open‐field apparatus consisting of a black square base (45 × 45 cm^2^) with black walls (45 cm in height). A single mouse was gently placed in the corner of the chamber, and after 1 min of adaptation, all spontaneous activities were recorded for 5 min using the video‐computerized tracking system. The total motion distance was used as an index of locomotion capacity.^45^

*Cytokines Analyses*: Levels of TNF‐α, IFN‐γ, IL‐6, IL‐10 in the serum and intestinal tissue of male mice were measured using Magnetic Luminex Assay (mice Catalog#LXSAMSM‐05 from R&D System, Minneapolis, MS, USA). All assays were analyzed on a Bio‐Plex MAGPIX Multiplex Reader (Bio‐Rad).

*Statistical Analysis*: Statistical analyses were carried out using SPSS version 18 (SPSS, Chicago, IL, US). All continuous variables such as bacterial α‐diversity, behavioral data, and age were presented as mean ± standard error of the mean (SEM) unless otherwise indicated and compared between groups using Student's *t*‐test. Categorical data (sex) were analyzed by Chi‐square test. Statistical significance level was set at *p* < 0.05.

*Attribution*: Portions of the Experimental Section and text were reprinted from our previous works,^17^, ^37^ with and without modifications, with permissions under a Creative Commons Attribution License 4.0 (CC BY) (https://creativecommons.org/licenses/by/4.0/),^17^ and under the authors' retained copyright,^37^ respectively.

## Conflict of Interest

The authors declare no conflict of interest.

## Supporting information

Supplementary
