# Supplementary material for: Perturbed Microbial Ecology in Myasthenia Gravis: Evidence from the Gut Microbiome and Fecal Metabolome
Source: Adv Sci (Weinh). 2019 Jul 31;6(18):1901441. doi: 10.1002/advs.201901441 (PMC6755540; doi:10.1002/advs.201901441)
Supplement: Supplementary file 1 — Supplementary [file ADVS-6-1901441-s001.pdf]

Copyright WILEY-VCH Verlag GmbH & Co. KGaA, 69469 Weinheim, Germany, 2019.

Supporting Information

for *Adv. Sci.,* DOI: 10.1002/advs.201901441

Perturbed Microbial Ecology in Myasthenia Gravis: Evidence from the Gut Microbiome and Fecal Metabolome

Peng Zheng, Yifan Li, Jing Wu, Hanping Zhang, Yu Huang, Xunmin Tan, Junxi Pan, Jiajia Duan, Weiwei Liang, Bangmin Yin, Fengli Deng, Seth W. Perry, Ma-Li Wong, Julio Licinio, Hong Wei, Gang Yu,* and Peng Xie**

Supporting Information

Figure. S1

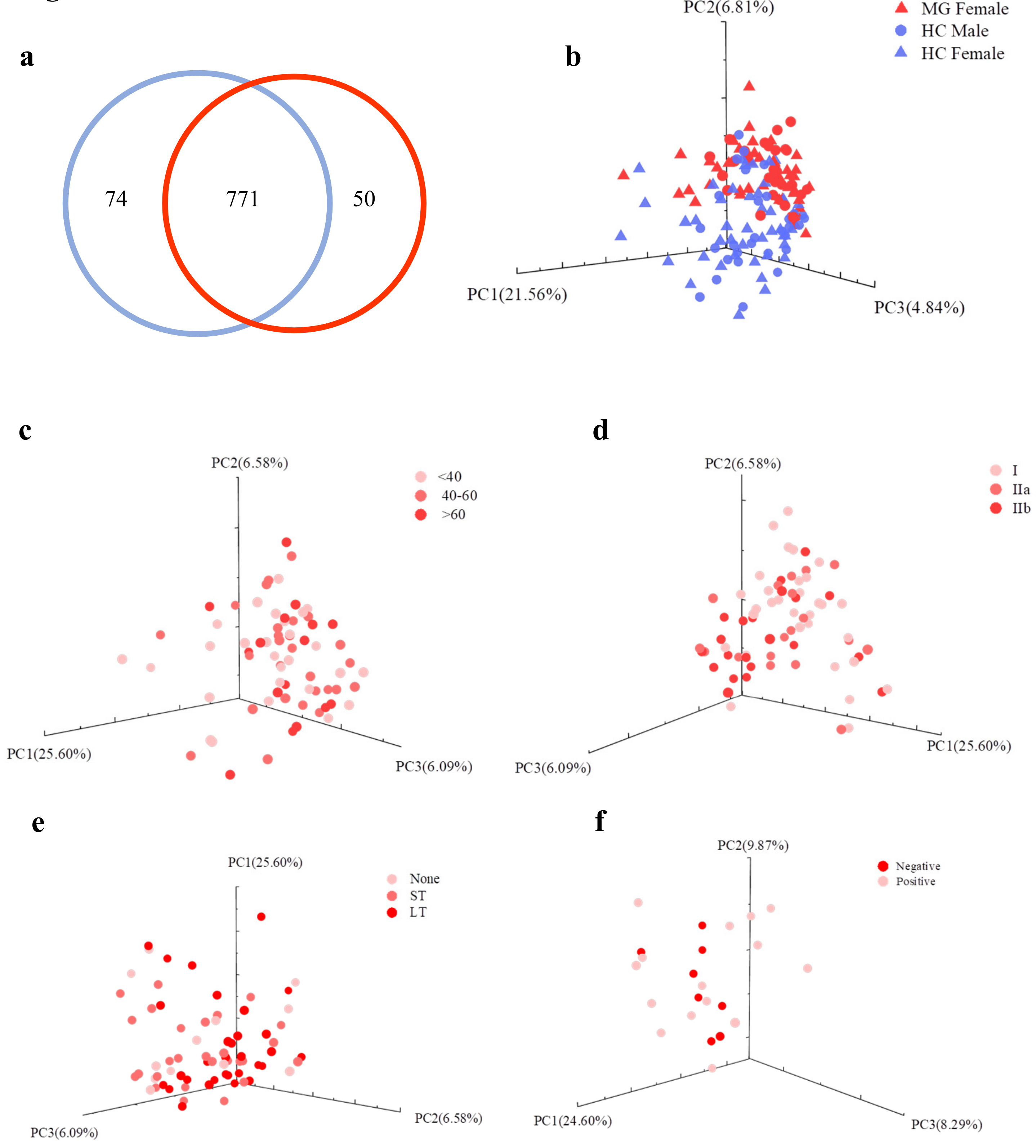

Figure. S1. (a) A Venn diagram showed that 771 of 895 OTUs were detected in the two groups, while 50 and 74 OTUs were unique to MG subjects (red circle, n=70) and HCs (blue circle, n=74), respectively. (b)The global microbial phenotypes were not clustered based on sex (n=44, HC Female; n=30, HC Male; n=39, MG Female; n=31, MG Male). (c-f) The global microbial phenotypes of MG groups were not clustered based on age (n=27, <40; n=26, 40-60; n=17, >60. MG), clinical classification, medication (n=30, LT; n=27, ST; n=12, None; LT: long term immunosuppressive treatment; ST: short term treatment; None: no treatment), and AChR antibody (n=15, Positive; n=8, Negative).

Figure. S2

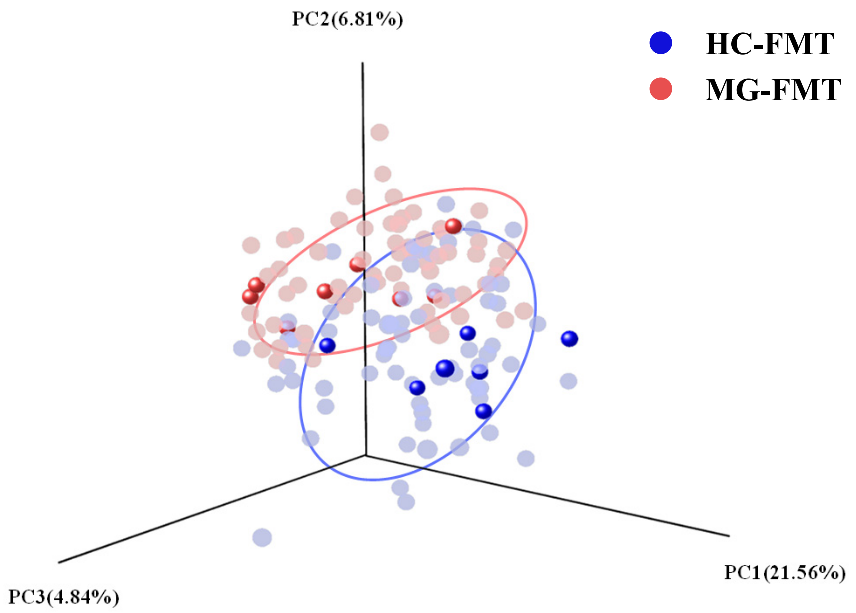

Figure. S2 The samples used for fecal microbiota transplantation experiments (n=7-8 for every group) were mixed with the remaining samples (n=74, HC; n=70, MG group).

Figure. S3

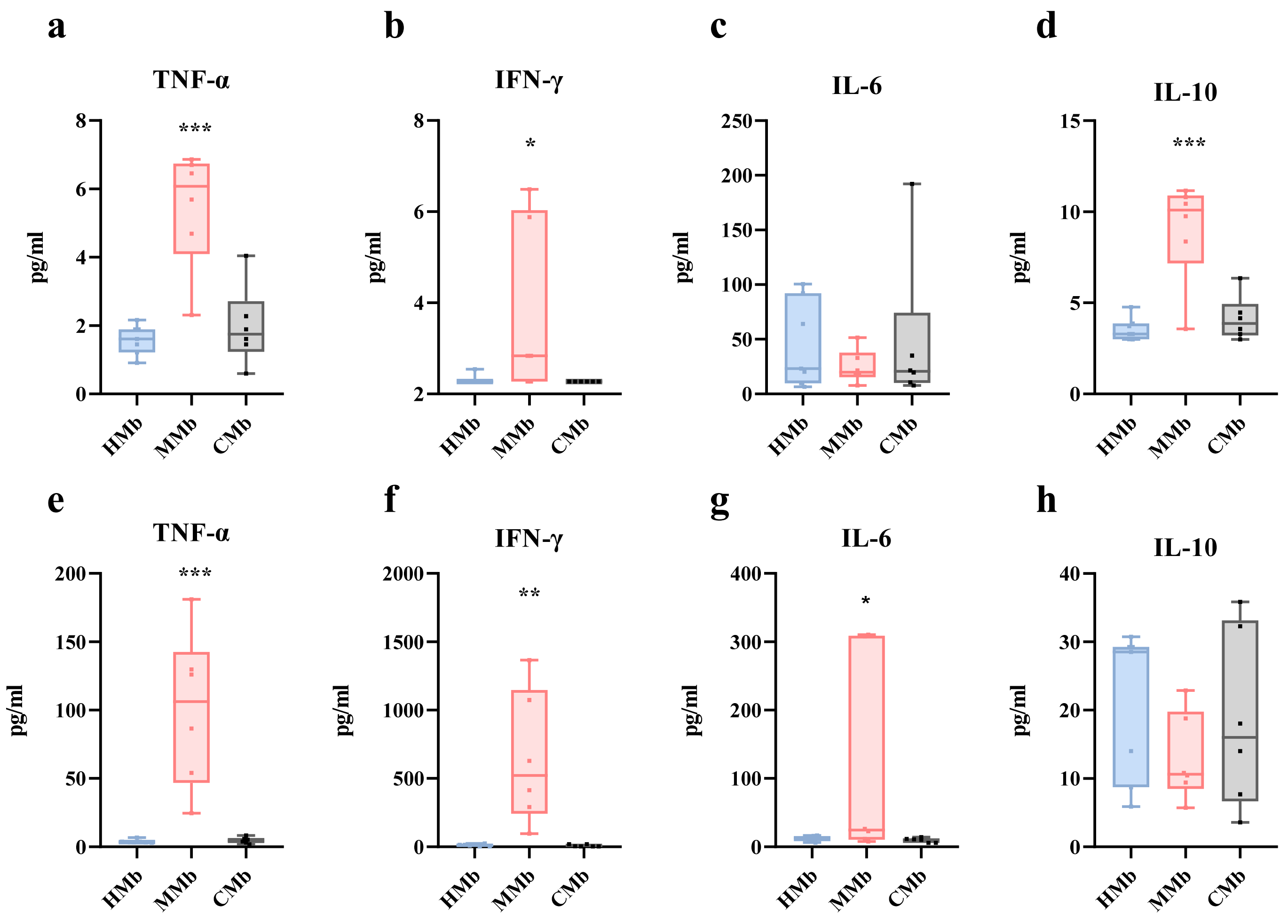

Figure. S3. The cytokine levels in serum and intestinal tissue among the three groups. (a-d) The serum levels of TNF-α, IFN-γ, and IL-10 were significantly increased in MMb group relative to HMb group. These increased cytokines can be reversed in CMb group. (e-h) Compared to HMb group, the levels of TNF-α, IFN-γ, and IL-6 were significantly un-regulated in MMb group; and these changes were reversed to normal level in CMb group. (male mice, HMb, n=7 ; MMb,n=6; CMb, n=6). * P<0.05, **P<0.01, ***P<0.001 by one way ANOVA .
